# Impact of malaria on haematological parameters of urban, peri-urban and rural residents in the Ashanti region of Ghana: a cross-sectional study

**DOI:** 10.12688/aasopenres.12979.3

**Published:** 2020-07-09

**Authors:** Abdul-Hakim Mutala, Kingsley Badu, Christian Owusu, Samuel Kekeli Agordzo, Austine Tweneboah, Dawood Ackom Abbas, Matthew Glover Addo

**Affiliations:** 1 Theoretical and Applied Biology, Kwame Nkrumah University of Science and Technology, Kumasi, Ashanti, Ghana; 2 Kumasi Center for Collaborative Research for Tropical Medicine, Kwame Nkrumah University of Science and Technology, Kumasi, Ashanti, Ghana

**Keywords:** malaria, anaemia, parasitaemia, WBC count, thrombocytopeania

## Abstract

**Background:** We aimed at investigating the impact of malaria on the haematological parameters of residents from different demographic settlements in the Ashanti Region of Ghana. Malaria parasites trigger changes in certain haematological parameters, which may result in a number of clinical manifestations. Differences in demographic settlements, such as rural, peri-urban and urban settlements may also influence these changes, but this has not been extensively studied in Ghana.

**Methods: **We conducted a hospital-based, cross-sectional study from January to December 2018 in three different settlements. A total of 598 participants were recruited. Blood smears were examined to detect and quantify malaria parasitaemia, while haematological parameters were measured using a haematology analyser.

**Results: **Participants from the rural settlement had the highest malaria prevalence (21.3%) compared to urban (11.8%) and peri-urban areas (13.3%); however, the peri-urban area had the highest median parasite density (568; IQR=190.0-1312.0). Age was significantly associated with the odds of malaria positivity (OR: 0.97; CI:0.96 — 0.99; *p*=4.96*10 ^-4^). When haematological parameters of the malaria-infected study participants were compared to the parameters of uninfected participants, red blood cell count (p=0.017), haemoglobin (p=0.0165), haematocrit (p=0.0015), mean corpuscular volume (p=0.0014), plateletcrit (p<0.0001) and platelet count (p<0.0001) were all significantly lower in the malaria infected group. In addition to age, haemoglobin and plateletcrit levels were also inversely correlated with the odds of testing positive for malaria, suggesting that children who were anaemic and/or thrombocytopaenic were likely to be infected. After fitting the data to a logistic regression model comprising the three variables, the model correctly categorised 78% of uninfected study participants, but only 50% of the malaria-positive participants.

**Conclusions: **Study participants who were positive for malaria were younger and had low haemoglobin and plateletcrit levels compared to uninfected individuals. Further studies are needed to more precisely elucidate the relationship between malaria infection,demographic and haematological parameters.

## Introduction

Malaria remains the most important protozoan infection of humans and continues to have an immense impact on the health and quality of life of people across the world. Despite the decrease in incidence of mortality due to malaria in the last decade, the most recent World Malaria Report revealed that 228 million clinical cases of malaria were reported, resulting in no less than 405,000 deaths, the majority of which were in sub-Saharan Africa ^1^.

The introduction of malaria parasites into the host peripheral blood by an infected female *Anopheles* mosquito triggers changes in several host haematological parameters, many of which play a role in malaria pathogenesis. These changes may subsequently affect the general physiology of the host, resulting in a number of clinical manifestations, with anaemia and thrombocytopaenia being the most common. Haematological parameters that are often affected include the relative numbers of circulating cell types such as erythrocytes, platelets, granulocytes and lymphocytes, as well as parameters like haemoglobin concentration ^2– 4^. While erythrocyte and platelet levels are consistently decreased in malaria-infected individuals, there have been conflicting reports on the effect of malaria on leukocyte counts. A recent study showed a significant reduction in leukocyte, as well as platelet and erythrocyte levels in malaria-infected study participants compared with their uninfected counterparts ^5^. Moreover, Kotepui *et al*. (2014) reported that low platelet, white blood cell (WBC) and lymphocyte counts were important predictors of malaria infection and, when used with other clinical methods, could improve malaria diagnosis and treatment ^6^. However, another study reported elevated leukocyte levels in malaria-infected study participants compared to their uninfected counterparts, suggesting that the relationship between malaria and certain immunohaematological parameters may be more complex than previously thought ^7^.

While haematological changes associated with malaria have been well-characterized ^6, 8, 9^, it is possible that factors such as differences in demographic settlements could also influence observed changes. However, there is relatively limited data on the differences in haematological indices of malaria-infected people in rural, peri-urban and urban settlements, especially in the forested zones. The aim of this study was to investigate the haematological indices of malaria-infected individuals across these different settlements.

## Methods

We carried out a cross-sectional study targeting hospital attendees. In order to avoid bias and avoid including only ‘sick' or symptomatic participants, we extended our sampling to include ‘healthy' participants who accompanied their relatives or friends to the hospital.

### Ethical statement

The protocol for data collection was reviewed and approved by the Committee on Human Research Publication and Ethics of the Kwame Nkrumah University of Science and Technology (CHRPE/KNUST) and the Komfo Anokye Teaching Hospital (CHRPE/KATH), (Ref: CHRPE AP/018/18). All study participants provided written informed consent prior to study enrolment, with parental or guardian consent obtained for children.

### Study sites

The study was conducted concurrently at the Kumasi South Hospital (KSH), the Kuntanase Government Hospital (KGH) and the Agona Government Hospital (AGH) in the Ashanti region of Ghana. KSH is located in Atonsu, a suburb of Kumasi, the regional capital and second largest city in Ghana, and served as the urban site. KGH is situated in Kuntanase, the capital of the Bosomtwe district. The Bosomtwe district is one of the 27 districts in the Ashanti region and is located approximately 28 kilometres from Kumasi. AGH is in the Sekyere East district and is located approximately 37 kilometres away from Kumasi. KGH and AGH served as the peri-urban and rural study sites, respectively. The urban, peri-urban and rural sites served as proxies for high, medium and low transmission transects. People in the Agona area receive over 11,000 mosquito bites annually, with 800 of them being infectious bites, while people in the Kumasi area receive approximately 400 bites and around 200 infectious bites per year. The transmission intensity in the Kuntanase area is between these two ^10, 11^.

### Study participants

The sample size was determined using the binomial model. Confidence intervals of 95% and a precision level of 5% was used. In the equation below, *n* is the sample size, *z* is the critical value of the standard normal distribution at 5% level (1.96), *p* is the estimated malaria prevalence, *q* = 1 – *p* and *d* is the precision level. The prevalence of malaria had previously been determined by Paintsil *et al.* (2019) to be about 26% ^12^.

$$n\;=\;{\text{z}}_{a}\text{pq}/{\text{d}}^{2}$$

The minimum sample size required was calculated to be 295; however, we sampled 601 participants to make up for the different transmission seasons (January to December 2018) across which samples were collected. The study targeted people accessing healthcare at the various hospitals. This included patients referred to the laboratory for malaria tests and accompanying caregivers who were not sick. The purpose of the study was explained to potential participants using a participant information leaflet to seek their informed consent, a copy of which is provided as *Extended data* ^13^. An interpreter was employed to translate the written document into the local dialect (Akan) for those who could not read. In addition, thumbprints were obtained for those who could not sign/write. Demographic data such as age, gender and insecticide-treated net (ITN) use were obtained from participants using a semi-structured questionnaire, a copy of which is provided as *Extended data* ^13^.

### Exclusion criteria

People who were critically ill (requiring hospitalisation) and those who declined to consent were excluded from the study. Infants under six months (<6 months) were also excluded.

### Haematological analysis

From each participant, 2 ml of venous blood was drawn by a trained phlebotomist in the hospital laboratory. This was transferred into an EDTA tube with a unique participant identifier. The blood sample was used to prepare thin and thick films for microscopic examination and automated complete blood counts (CBCs). Blood parameters were estimated using the Sysmex XP-300 Automated Haematology Analyzer. The cell counter provided data on red blood cell (RBC) count, haemoglobin (Hb) level, mean corpuscular volume (MCV), mean corpuscular haemoglobin (MCH) level, WBC, lymphocyte, neutrophil, platelet counts, mean platelet volume (MPV), plateletcrit and the red blood cell distribution width (RDW).

### Parasitological analysis

From the EDTA-treated blood, 6 µL and 3 µL were used to prepare thick and thin blood films, respectively. After air-drying, the thin blood smears were fixed with methanol and both smears subsequently stained with 10% Giemsa for 15 minutes. Two experienced microscopists independently examined the slides under ×100 oil immersion to determine the presence or absence of malaria parasites. Parasites were quantified after counting 200 or 500 WBCs. Parasite densities were calculated as parasite per microliter of blood (parasite counted / WBCs counted × total WBC in 1µL of blood). A slide was only declared negative when no malaria parasite was seen after scanning 100 high power fields (HPFs).

### Data collection technique

The data were gathered by the authors using standardized questionnaire. At the end of each day, all researchers came together to check the completeness of each individual questionnaire and match them to the lab specimens taken. Prior to the survey we had pre-tested the questionnaire in a community setting to ensure that people understood the questions. Feedback were used to improve the questionnaire before the actual survey was carried out.

### Statistical analysis

The data collected were coded and entered into Microsoft Excel 2016. The data were checked for completeness. Samples with missing data were excluded from the analysis. Thus, three samples with missing microscopy data were excluded completely from all analyses. Data analysis was performed using GraphPad Prism v6 (GraphPad Software, Inc., San Diego, CA, USA). Data normality was checked using the Shapiro-Wilk normality test. For normally distributed data, comparisons were carried out using one-way ANOVA, whilst data not conforming to the normal distribution were compared using the Kruskal-Wallis or Mann-Whitney Tests. Pairwise multiple comparison across communities was done using Dunn’s multiple comparison test. For the logistic regression analyses, 19 samples were excluded due to incomplete data, bringing the sample count to 579. The regression analyses were carried out using the *glm* function in R-3.6.2 ^14^. Results were considered statistically significant if *p* ≤ 0.05.

## Results

In total, 598 participant samples were examined for parasite prevalence and density. A further 19 samples were excluded from the haematological analysis because the haematological indices could not be determined. Table 1 gives a summary of the demographic profile of the study population.

**Table 1.** Baseline characteristics of participants and study areas.

Baseline characteristics	*n* (%)	*P value*
***Age(yrs)***		
0–5 Urban Peri-urban Rural	11(19.6) 16(28.6) 29(51.8)	0.006
6–14 Urban Peri-urban Rural	8(21.1) 8(21.1) 22(57.8)	0.004
15+ Urban Peri-urban Rural	185(36.7) 171(33.9) 149(29.5)	<0.0001
***Gender(Female)***		
Urban Peri-urban Rural	161(78.9) 150(76.9) 139(65.5)	0.070
***ITN usage***		
Urban Peri-urban Rural	66(32.3) 57(29.2) 60(30.3)	0.7932
***Settlement***		
Urban Peri-urban Rural	203 (33.9) 195 (32.6) 200 (33.4)	0.884
***Malaria prevalence***		
Urban Peri-urban Rural	24 (11.8) 26 (13.3) 43 (21.3)	<0.0001

The results were considered significant if p ≤ 0.05

Out of the 598 samples analysed in the study, 75.4% ( *n*=451) were female ^13^. The overall median age was 27 years ( *IQR*=19–40) and there was a statistically significant difference in the age profiles of participants from the three study sites ( *H=* 20.8; *p*<0.0001). Study participants in the rural area were comparatively younger, with a median age of 24.5 years ( *IQR*=14–35), followed by those in the peri-urban area, with a median age of 27 years ( *IQR*=19.5–41.5) and finally those in the urban area, with a median age of 29 years ( *IQR*=22.5–43.0). This observation is consistent with expectations as, typically, the youth tend to leave their rural communities in search of jobs as they get older.

**Figure 1.**
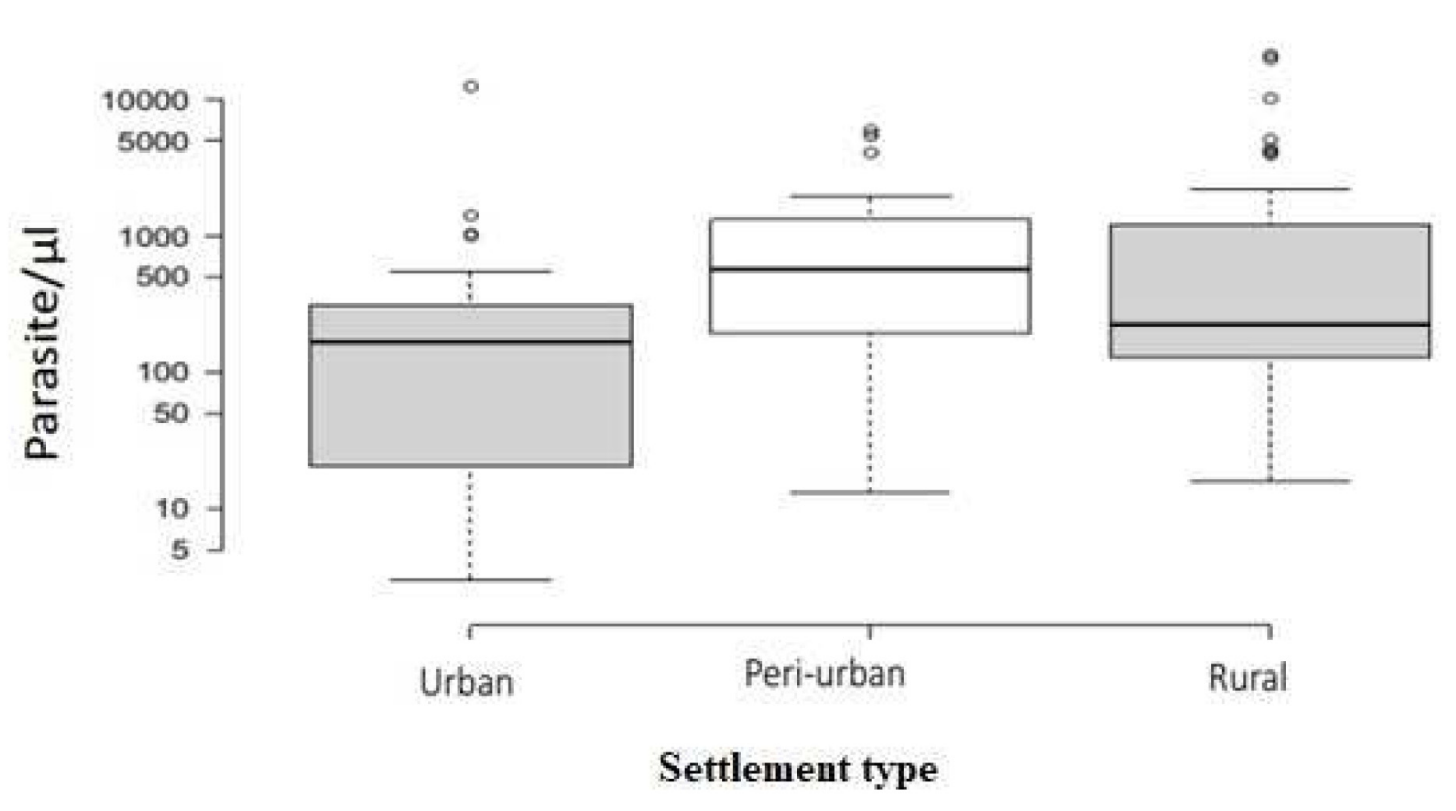
Parasite density across the three types of settlements. Centre lines show the median counts; box limits indicate the 25th and 75th percentiles as determined by R; whiskers extend 1.5 times the interquartile range from the 25th and 75th percentiles. Widths of boxes are proportional to square-roots of the number of observations, Urban=24, Peri-urban=26 and Rural=43 sample points. The Y-axis shows the estimated number of parasites per microlitre in blood in logarithmic scale. The results were considered significant if *p* ≤ 0.05.

16% ( *n*=93) of the study participants had *Plasmodium falciparum* infection, confirmed by microscopy. Children between the ages of six and 14 (inclusive) recorded both the highest rates of malaria prevalence (34.8%) and the lowest rates of ITN usage (28.9%) while children less than five years constituted the majority of those who slept under insecticide-treated mosquito nets (58.9%). There was a significant difference in the median parasite densities of infected study participants across the different age groups ( *H*=8.64; *p*=0.0133). Children under five years harboured the highest number of parasites, with a median parasite density of 665 *(IQR*=327–1038), followed by children 6–14 years, with a median density of 504 ( *IQR*=160–4139), and study participants ≥15 years, with a median density of 24.5 ( *IQR*=13–40). Study participants from the urban area reported the highest ITN usage (32.3%; *n*=66/204), with people from the peri-urban (29.2%; *n*=57/195) and rural areas (30.3%; *n*=61/202) reporting slightly lower percentages of usage ( *p*=0.7932). In this case, however, the highest malaria prevalence was recorded in rural inhabitants (21.3%; *n*=43/202), followed by the peri-urban area (13.3%; *n*=26/195), with the urban centre recording the lowest prevalence (11.8%, *n*=24/204). There was also a significant difference in the parasite densities of infected participants across the three communities ( *H*=8.41; *p*=0.0149). Study participants in the peri-urban area recorded the highest median parasite density (568; *IQR*=190–1312), followed by the rural area (224; *IQR*=126–1198), with the urban area recording the lowest median parasite density (167; *IQR*=20.5–311.5) ( Figure 1).

### Age is a significant demographic predictor of malaria infection status

To test the relationship between the various demographic parameters and infection status, we carried out univariate logistic regression analyses of each predictor against the response variable. In order to avoid discarding potentially significant variables, we used a relaxed significance threshold of *p* ≤ 0.25. All the demographic predictors passed this threshold with the exception of ITN usage ( *p*=0.838), which was subsequently discarded. The remaining variables were used to create a multivariate logistic model, with age emerging as the only significant predictor of infection status ( Table 2).

**Table 2.** The association between malaria infection status and age is independent of gender and settlement type.

Predictor	Coeff. est.	Std. error	Z-value	OR (95% CI)	p-value
Intercept	-0.70	0.27	-2.56	0.50 (0.29 — 0.85)	**0.010494**
Sex: M	0.11	0.27	0.42	1.12 (0.66 — 1.88)	0.675678
**Age**	-0.03	0.01	-3.48	0.97 (0.96 — 0.99)	**0.000496**
Community: Peri-urban	-0.47	0.28	-1.67	0.63 (0.36 — 1.08)	0.094920
Community:Urban	-0.52	0.29	-1.82	0.59 (0.34 — 1.04)	0.068121

Results were considered significant if *p* ≤ 0.05.

### Malaria causes significant changes in several haematological parameters

The median haematological parameters of the infected and non-malaria groups were compared using the Mann-Whitney test due to the non-parametric distribution of the data. There were no significant differences in the median counts of neutrophils, lymphocytes or WBCs between the infected and non-infected groups; however, individuals with malaria had significantly lower median values of RBCs ( *p*=0.017), Hb ( *p*=0.0165), haematocrit ( *p*=0.0015), MCV ( *p*=0.00139), platelets ( *p*<0.0001) and plateletcrit ( *p*<0.0001) compared to malaria-negative individuals ( Table 3).

**Table 3.** Levels of haematological parameters in infected vs uninfected study participants.

Variable	Malaria-infected	Uninfected	P-value
	*Median (IQR)*	*Median (IQR)*	
Red blood cells (RBC) (×10 ^6^ /µL) ^a^	4.16 (3.78 – 4.67)	4.42 (3.93 – 4.85)	**0.0170**
Haemoglobin (g/dL) ^a^	11.10 (9.5 – 12.4)	11.60 (10.40 – 12.80)	**0.0165**
Haematocrit (%) ^a^	39.30 (34.40 – 44.60)	42.80 (38.20 – 46.20)	**0.0015**
Mean corpuscular volume (MCV) (fL) ^a^	94.20 (89.3 – 100.5)	97.80 (91.70 – 102.70)	**0.00139**
Mean corpuscular haemoglobin (MCH) (pg) ^a^	26.60 (25.2 – 28.0)	26.80 (24.80 – 28.50)	0.5413
Red cell distribution width (RDW-SD) (fL) ^a^	50.90 (47.40 – 57.10)	51.00(47.70 – 57.40)	0.7782
Red cell distribution width (RDW-CV) (%) ^a^	14.30 (14.30 – 15.90)	13.80 (12.60 – 15.80)	0.1482
Platelet (×10 ^3^/µL) ^a^	128.00 (72.0 – 186.0)	172 (119.0 – 229.0)	**<0.0001**
Plateletcrit (PCT) (%) ^a^	0.15 (0.08 – 0.22)	0.20 (0.13 – 0.26)	**<0.0001**
Platelet distribution width (PDW) (fL) ^a^	16.30 (14.30 – 19.60)	16.20 (13.60 – 19.05)	0.1544
Mean platelet volume (MPV) (fL) ^a^	11.20 (10.20 – 12.30)	11.20 (10.10 – 12.20)	0.9878
Platelet large cell ratio (P-LCR) (%) ^a^	35.70 (29.40 – 43.0)	35.50 (28.30 – 43.90)	0.7465
White blood cells (WBC) (×10 ^3^/µL) ^b^	3.50 (2.20 – 5.35)	3.50 (2.40 – 4.80)	0.5078
Neutrophil (×10 ^3^/µL) ^a^	1.00 (0.50 – 2.10)	1.00 (0.70 – 1.70)	0.9702
Lymphocyte (×10 ^3^/µL) ^a^	1.80 (1.20 – 3.0)	2.20 (1.50 – 3.10)	0.8222

^a^T-test; ^b^Man-Whitney U; IQR=Interquartile range

Youth, anaemia and thrombocytopaenia are associated with increased odds of malaria positivity

To determine key predictors of infection status, we created multivariate models using the six significant haematological parameters from Table 2, as well as the only significant demographic variable: age ( Table 3). Model selection was done using the forward stepwise regression method. As some of the haematological parameters were highly correlated with each other (RBC, HB and HCT; PLT and PCT), we decided to only select the correlated variables with the largest effect sizes as inclusion of correlated predictors reduces the precision of the estimated coefficients and inflates estimates of the standard error. The analysis identified age ( *p*=2.06*10 ^-5^), haemoglobin level ( *p*=0.046) and plateletcrit ( *p*=0.0001) as being significantly inversely associated with malaria positivity ( Table 4). As all three predictors are continuous variables, care must be taken when interpreting the estimated coefficients. Applying the exponential function to the estimated coefficient of a continuous variable ( *e* ^β^) produces the odds ratio associated with unitary increments of the variable.

**Table 4.** Demographic and haematological predictors of malaria infection status.

Predictor	Coeff. est.	Std. error	Z-value	OR (95% CI)	p-value
Intercept	1.45	0.66	2.19	4.264 (1.162 — 15.656)	**0.028841**
HGB	-0.11	0.06	-2.00	0.894 (0.801— 0.997)	**0.045799**
Age	-0.03	0.01	-4.26	0.966 (0.951— 0.981)	2.06*10 ^-5^
PCT	-5.03	1.32	-3.81	0.006 (0.0004 — 0.0869)	**0.000139**

The results were considered significant if p ≤ 0.05.

Anaemia and thrombocytopaenia comprise two of the most common complications associated with malaria. Anaemia and acute anaemia are defined as having Hb levels <11g/dl or <5g/dl, respectively ^15^, while thrombocytopaenia is defined as platelet count<150×10 ^3^/μL ^16^. 48.4% ( *n*=45/93) of all malaria-infected individuals in this study were anaemic compared to 33.3% ( *n*=164/492) of the control group ( *p*=0.0054), with two malaria-infected participants matching the criteria for acute anaemia. In addition, 58.1% ( *n*=54/93) of participants from the infected group were thrombocytopaenic.

While all the variables included in the final model are statistically significant, that does not necessarily mean that the model is a good fit for the data. As logistic regression deals with odds and probabilities, we can use the model to predict the probability of being malaria-positive. In order to map our logistic regression values to one of the two response categories (infected or uninfected), we first defined a decision threshold ( *p*>0.21) that balanced model sensitivity with specificity, and made our predictions based on this threshold. The model correctly identified 78.4% of malaria-negative individuals but only 50% those who were positive ( Table 5).

**Table 5.** Confusion matrix comparing the actual outcomes to the predicted outcomes.

	Negative	Positive
**Pred. Neg.**	382	46
**Pred. Pos.**	105	46

The predictions were classified using a decision threshold of *p* > 0.21

## Discussion

We investigated the effect of malaria on haematological parameters of people in three different demographic settlements: urban, peri-urban and rural communities. By 2050, it is predicted that 58% of people in sub-Saharan Africa will be living in urban areas, compared with approximately 40% currently ^17^. This is expected to have a significant impact on the prevalence and clinical outcomes of infectious diseases like malaria as the increasing urban population further widens urban-rural economic and resource divides. In this study, people from the rural area recorded the highest prevalence of malaria compared to people from the urban and peri-urban areas, a finding that is consistent with results from other studies conducted in Ghana ^18, 19^. Rural areas are often described as intense and perennial transmission areas as there are often several suitable *Anopheles* breeding sites available, coupled with poor access and/or adherence to vector control measures by rural inhabitants ^20, 21^. The relatively younger age observed in the rural area may also be a contributing factor to the high prevalence observed in this study area, as children represent a high risk group for malaria infection ^22^. In line with this, the majority of infected individuals in the rural area were children less than five years. Children under five years may not have a fully developed immune system and are, therefore, more susceptible to infections. Adults, on the other hand, have relatively stronger immune systems and often have partial immunity to malaria from previous exposure ^23^. This might explain why the older generation recorded the lowest parasite densities.

Consistent with findings from other studies ^2, 6, 18^, this study also found significant differences in several red blood cell parameters between malaria-infected and uninfected study participants. *Plasmodium falciparum*, the parasite that causes the most severe form of malaria in humans, invades and multiplies inside red blood cells in a destructive cycle that is responsible for much of the severity and mortality associated with the disease ^24, 25^. Haemolytic mechanisms are usually employed by the host immune system to eliminate parasitized red blood cells in a process that may lead to anaemia ^26, 27^. Anaemia is considered to be one of the most common complications of malaria, especially in children and pregnant women ^28^. The present study revealed significantly lower Hb levels in the infected population compared to the uninfected group ( Table 3); however, a substantial proportion of the uninfected study participants also met the criteria for anaemia. The suspected anaemia cases observed in the control population may be due in part to poor nutritional status, undetectable malaria infection or, to a lesser extent, helminth infections ^2^. Peripheral leukocyte or WBC counts have also been noted as being in the low to normal range during malaria, a phenomenon which is counterintuitive as one would expect increased production of WBCs during infection. Malaria-infected individuals have been reported to have significantly lower lymphocyte levels likely as a result of their withdrawal from the peripheral circulation and sequestration in lymph tissue, rather than actual depletion of the cell population ^29^. However, there are reports of both increased (leukocytosis) ^4^ and decreased (leucopaenia) ^2, 7^ WBC levels in malaria-infected individuals. The present study did not observe any significant changes in the WBCs levels of participants with malaria compared to the uninfected group. One of the most striking results from the study was the low levels of platelet and plateletcrit (a measure of total platelet mass) observed in malaria-infected study participants compared to uninfected participants, a finding that is consistent with results from other studies ^6, 7, 16^. This observation is particularly common in *Plasmodium falciparum* ^4, 30^ and *Plasmodium vivax* infections ^31, 32^. In this present study, 58.1% of malaria-infected individuals were thrombocytopaenic. There are several hypotheses about the reduction of platelets during malaria infection. This abnormality may be the result of blood clots developing in the bloodstream, thereby blocking small blood vessels. The intermittent clotting subsequently depletes the number of circulating platelets in the bloodstream ^33^. The reduction in platelet levels may also be attributable to an immune-mediated mechanism, whereby specific immunoglobulin G (IgG) produced as a result of the parasite invasion, forms a complex with parasite antigens. The resulting complex then binds to and damages platelets, with damaged platelets subsequently removed from circulation ^33, 34^. Ultimately, plateletcrit was the strongest predictor of infection status, with 99.4% increased odds of being malaria-positive for each unit increase in plateletcrit levels. In spite of this, the full predictive model performed rather poorly as only 50% of infected individuals were correctly classified as such. Further studies are needed to more precisely elucidate the relationship between malaria infection status and various host demographic and haematological parameters. Limitations of this study included a skewed gender-balance and a lack of information on recent medical histories as there are many other diseases and conditions that may affect haematological values and could potentially affect the interpretation of the results.

## Data availability

### Underlying data

Harvard Dataverse: Replication Data for: Impact of malaria on haematological parameters of urban, peri-urban and rural patients in the Ashanti region of Ghana. https://doi.org/10.7910/DVN/NGBMZM ^13^

This project contains the following underlying data:

- Combined data.xlsx (raw demographic, haematological and parasitological data for all participants)
- Combined Data_Age.tab (raw demographic, haematological and parasitological data for all participants, including ages)
- Malaria Infected population.xlsx (raw demographic, haematological and parasitological data for malaria infected participants)
- Malaria Uninfected.xlsx (raw demographic, haematological and parasitological data for uninfected participants)
- Peri-Urban population.xlsx (raw demographic, haematological and parasitological data for participants in the peri-urban area)
- Rural population.xlsx (raw demographic, haematological and parasitological data for participants in the rural area)
- Urban population.xlsx (raw demographic, haematological and parasitological data for participants in the urban area)
- Data Dictionary.xlsx

### Extended data

Harvard Dataverse: Replication Data for: Impact of malaria on haematological parameters of urban, peri-urban and rural patients in the Ashanti region of Ghana. https://doi.org/10.7910/DVN/NGBMZM ^13^

This project contains the following extended data:

- CHRPE Participant Information Leaflet_Malaria.pdf
- Questionnaire- Malaria.pdf

Data are available under the terms of the Creative Commons Zero "No rights reserved" data waiver (CC0 1.0 Public domain dedication).

## References

[1] World Health Organization: World malaria report 2018. World Health Organization;2018 Reference Source

[2] Maina RN Walsh D Gaddy C: Impact of *Plasmodium falciparum* infection on haematological parameters in children living in Western Kenya. *Malar J.* 2010;9 Suppl 3 (Suppl 3):S4. 10.1186/1475-2875-9-S3-S4 21144084 PMC3002140

[3] Waitumbi JN Opollo MO Muga RO: Red cell surface changes and erythrophagocytosis in children with severe *plasmodium falciparum* anemia. *Blood.* 2000;95(4):1481–1486. 10666228

[4] Erhart LM Yingyuen K Chuanak N: Hematologic and clinical indices of malaria in a semi-immune population of western Thailand. *Am J Trop Med Hyg.* 2004;70(1):8–14. 14971691

[5] Bakhubaira S: Hematological Parameters in Severe Complicated *Plasmodium falciparum* Malaria among Adults in Aden. *Turk J Haematol.* 2013;30(4):394–399. 10.4274/Tjh.2012.0086 24385830 PMC3874965

[6] Kotepui M Phunphuech B Phiwklam N: Effect of malarial infection on haematological parameters in population near Thailand-Myanmar border. *Malar J.* 2014;13:218. 10.1186/1475-2875-13-218 24898891 PMC4053303

[7] Ladhani S Lowe B Cole AO: Changes in white blood cells and platelets in children with falciparum malaria: relationship to disease outcome. *Br J Haematol.* 2002;119(3):839–847. 10.1046/j.1365-2141.2002.03904.x 12437669

[8] Kotepui M Piwkham D PhunPhuech B: Effects of malaria parasite density on blood cell parameters. *PLoS One.* 2015;10(3):e0121057. 10.1371/journal.pone.0121057 25807235 PMC4373695

[9] Anabire NG Aryee PA Helegbe GK: Hematological abnormalities in patients with malaria and typhoid in Tamale Metropolis of Ghana. *BMC Res Notes.* 2018;11(1):353. 10.1186/s13104-018-3456-9 29871667 PMC5989466

[10] Abonuusum A Owusu-Daako K Tannich E: Malaria transmission in two rural communities in the forest zone of Ghana. *Parasitol Res.* 2011;108(6):1465–1471. 10.1007/s00436-010-2195-1 21153839

[11] Basing AW Tay S: Malaria transmission dynamics of the Anopheles mosquito in Kumasi, Ghana. 16th ICID Abstracts / International Journal of Infectious Diseases 21S. 2014;1–460. 10.1016/j.ijid.2014.03.456

[12] Paintsil EK Omari-Sasu AY Addo MG: Analysis of Haematological Parameters as Predictors of Malaria Infection Using a Logistic Regression Model: A Case Study of a Hospital in the Ashanti Region of Ghana. *Malar Res Treat.*hindawi.com;2019; 1486370. 10.1155/2019/1486370 PMC6556344 31263541

[13] Badu K: Replication Data for: Impact of malaria on haematological parameters of urban, peri-urban and rural patients in the Ashanti region of Ghana. Harvard Dataverse, V1.2019 10.7910/DVN/NGBMZM PMC7355218 32704620

[14] R Core Team: R: A language and environment for statistical computing. R Foundation for Statistical Computing, Vienna, Austria.2019 Reference Source

[15] World Health Organization: Haemoglobin concentrations for the diagnosis of anaemia and assessment of severity. World Health Organization; Report No.: WHO/NMH/NHD/MNM/11.1.2011 Reference Source

[16] Lacerda MV Mourão MP Coelho HC: Thrombocytopenia in malaria: who cares? *Mem Inst Oswaldo Cruz.* 2011;106 Suppl 1:52–63. 10.1590/s0074-02762011000900007 21881757

[17] United Nations, Department of Economic and Social Affairs, Population Division: World Urbanization Prospects: The 2018 Revision.2018[cited 27 May 2018]. Reference Source

[18] Iqbal SA Botchway F Badu K: Hematological Differences among Malaria Patients in Rural and Urban Ghana. *J Trop Pediatr.* 2016;62(6):477–486. 10.1093/tropej/fmw038 27318111 PMC5141942

[19] Ronald LA Kenny SL Klinkenberg E: Malaria and anaemia among children in two communities of Kumasi, Ghana: a cross-sectional survey. *Malar J.* 2006;5:105. 10.1186/1475-2875-5-105 17094806 PMC1654171

[20] Molina Gómez K Caicedo MA Gaitán A: Characterizing the malaria rural-to-urban transmission interface: The importance of reactive case detection. *PLoS Negl Trop Dis.* 2017;11(7):e0005780. 10.1371/journal.pntd.0005780 28715415 PMC5531679

[21] Trape JF Zoulani A: Malaria and urbanization in central Africa: the example of Brazzaville. Part II: Results of entomological surveys and epidemiological analysis. *Trans R Soc Trop Med Hyg.* 1987;81 Suppl 2:10–18. 10.1016/0035-9203(87)90472-x 2901796

[22] Vorasan N Pan-Ngum W Jittamala P: Long-term impact of childhood malaria infection on school performance among school children in a malaria endemic area along the Thai--Myanmar border. *Malar J.*BioMed Central;2015;14:401 10.1186/s12936-015-0917-7 26453016 PMC4600307

[23] Weng NP: Aging of the immune system: how much can the adaptive immune system adapt? *Immunity.* 2006;24(5):495–499. 10.1016/j.immuni.2006.05.001 16713964 PMC2266981

[24] Mkumbaye SI Wang CW Lyimo E: The Severity of *Plasmodium falciparum* Infection Is Associated with Transcript Levels of *var* Genes Encoding Endothelial Protein C Receptor-Binding *P. falciparum* Erythrocyte Membrane Protein 1. *Infect Immun.* 2017;85(4): pii: e00841-16. 10.1128/IAI.00841-16 28138022 PMC5364309

[25] Haldar K Mohandas N: Malaria, erythrocytic infection, and anemia. *Hematology Am Soc Hematol Educ Program.* 2009;87–93. 10.1182/asheducation-2009.1.87 20008186 PMC2933134

[26] Weatherall DJ Miller LH Baruch DI: Malaria and the red cell. *Hematology Am Soc Hematol Educ Program.* 2002;35–57. 10.1182/asheducation-2002.1.35 12446418

[27] Price RN Simpson JA Nosten F: Factors contributing to anemia after uncomplicated falciparum malaria. *Am J Trop Med Hyg.* 2001;65(5):614–622. 10.4269/ajtmh.2001.65.614 11716124 PMC4337986

[28] Menendez C Fleming AF Alonso PL: Malaria-related anaemia. *Parasitol Today.* 2000;16(11):469–476. 10.1016/S0169-4758(00)01774-9 11063857

[29] McKenzie FE Prudhomme WA Magill AJ: White blood cell counts and malaria. *J Infect Dis.* 2005;192(2):323–330. 10.1086/431152 15962228 PMC2481386

[30] Wickramasinghe SN Abdalla SH: Blood and bone marrow changes in malaria. *Baillieres Best Pract Res Clin Haematol.* 2000;13(2): 277–299. 10.1053/beha.1999.0072 10942626

[31] Oh MD Shin H Shin D: Clinical features of vivax malaria. *Am J Trop Med Hyg.* 2001;65(2):143–146. 10.4269/ajtmh.2001.65.143 11508390

[32] Lee HK Lim J Kim M: Immunological alterations associated with *Plasmodium vivax* malaria in South Korea. *Ann Trop Med Parasitol.* 2001;95(1):31–39. 10.1080/00034980020035898 11235551

[33] Essien EM: The circulating platelet in acute malaria infection. *Br J Haematol.* 1989;72(4):589–590. 10.1111/j.1365-2141.1989.tb04329.x 2673332

[34] Moulin F Lesage F Legros AH: Thrombocytopenia and *Plasmodium falciparum* malaria in children with different exposures. *Arch Dis Child.* 2003;88(6):540–541. 10.1136/adc.88.6.540 12765928 PMC1763122

