# Supplementary material for: Impact of malaria on haematological parameters of urban, peri-urban and rural residents in the Ashanti region of Ghana: a cross-sectional study
Source: AAS Open Res. 2020 Jul 9;2:27. Originally published 2019 Jul 29. [Version 3] doi: 10.12688/aasopenres.12979.3 (PMC7355218; doi:10.12688/aasopenres.12979.3)
Supplement: Peer review history

# Peer review: Reviewer response for version 3

**DOI:** 10.21956/aasopenres.14205.r27538

**Published:** 2020-07-10

**Authors:** Kwadwo Asamoah Kusi

**Affiliations:** 1 Department of Immunology, Noguchi Memorial Institute for Medical Research (NMIMR), University of Ghana, Legon, Ghana

The authors have made significant improvements based on the last review, but remnants of the requested portions needing review remain. For example, the concluding statement of the abstract is expected to directly respond to the study object of comparing haematological indices across the three sites, but it still reports comparison of infected and uninfected persons.

 Wherever this infected/uninfected comparison is made, it is not clarified whether it is for the entire data set from the three sites, or a per site analysis. These are minor issues that will greatly enhance the paper as it is.

Is the work clearly and accurately presented and does it cite the current literature?

Partly

If applicable, is the statistical analysis and its interpretation appropriate?

Partly

Are all the source data underlying the results available to ensure full reproducibility?

Partly

Is the study design appropriate and is the work technically sound?

No

Are the conclusions drawn adequately supported by the results?

No

Are sufficient details of methods and analysis provided to allow replication by others?

Partly

Reviewer Expertise:

Immunology of infectious diseases, vaccine discovery and development

I confirm that I have read this submission and believe that I have an appropriate level of expertise to confirm that it is of an acceptable scientific standard.

# Peer review: Reviewer response for version 2

**DOI:** 10.21956/aasopenres.14189.r27489

**Published:** 2020-06-16

**Authors:** Deus S. Ishengoma

**Affiliations:** 1 Tanga Research Centre, National Institute for Medical Research (NIMR), Tanga, Tanzania

**Abstract: **

- The following statement; “Age was significantly associated with the odds of malaria positivity (OR: 0.97; CI:0.96 — 0.99)” should have p-value. Despite a narrow confidence interval, the OR is almost 1.0 which suggests that the association cannot be significantly different. Based on table 2, I still recommend to report adjusted OR rather than crude OR which can potentially suffer from bias and confounding.

**Introduction:**

- In the second statement, “Despite the decrease in incidence of mortality due to malaria in the last decade, the most recent World Malaria Report revealed that **two hundred and twenty-eight million** clinical cases of malaria were reported, resulting in no less than 405,000 deaths, the majority of which were in sub-Saharan Africa”, the number of cases should be written in figures (i.e 228 million).

**Methods:**

- The authors stated that, “Babies younger than six months, patients who were critically ill and those dissenting to give their consent were excluded from the study”. However, babies (<6 months old) are not mentioned in the revised manuscript. This should be corrected.
- I would recommend to add a separate sub-section on collection of demographic data. The details provided in the responses to my previous comments should be summarized and incorporated in the manuscript. “ *The data, that is to say, the questionnaire were administered by the authors of this article and not by research assistants. Authors are very experienced in conducting similar surveys from different parts of the country and in other research projects. At the end of everyday all researchers came together to check the completeness of each individual questionnaire and match them to the lab specimen taken. Prior to the survey we had pre-tested the questionnaire in a community setting to ensure people understood and that questions were effective. Feedback were used to improve the questionnaire before the actual survey was carried out."*

**Results:**

- For the following statement, “Study participants from the urban area reported the highest ITN usage (32.3%; *n*=66/204), with people from the peri-urban (29.2%; *n*=57/195) and rural areas (30.3%; *n*=61/202) reporting significantly lower percentages of usage”; please provide a p-value.

Is the work clearly and accurately presented and does it cite the current literature?

Partly

If applicable, is the statistical analysis and its interpretation appropriate?

No

Are all the source data underlying the results available to ensure full reproducibility?

Partly

Is the study design appropriate and is the work technically sound?

No

Are the conclusions drawn adequately supported by the results?

Partly

Are sufficient details of methods and analysis provided to allow replication by others?

No

Reviewer Expertise:

Antimalarial drug resistance and Genomic epidemiology of malaria

I confirm that I have read this submission and believe that I have an appropriate level of expertise to confirm that it is of an acceptable scientific standard.

# Peer review: Reviewer response for version 2

**DOI:** 10.21956/aasopenres.14189.r27488

**Published:** 2020-06-08

**Authors:** Kwadwo Asamoah Kusi

**Affiliations:** 1 Department of Immunology, Noguchi Memorial Institute for Medical Research (NMIMR), University of Ghana, Legon, Ghana

- The authors have not provided the ethics approval number and also not indicated exclusion criteria that are likely to affect haematological parameters. E.g. being immunocompromised through HIV infection can affect haematological indices. Acknowledging the fact however, that authors have identified this as a study limitation.
- Table 1 should be re-organized to present information for each of the three hospitals used, and include a column with statistical analysis outcomes (p values) comparing parameters amongst the three sites. Analysis of the entire data set should follow this format, since comparing the measured parameters across the three transmission zones is what the study set out to do. I see from the tracked changes version that such results comparing the three sites were initially presented, but have now been removed.
  Knowing which parameters are different in Table 1 will be a useful guide for identifying potential confounders for regression analysis. 
- The analysis should compare the different haematological, demographic and clinical parameters between the three sites. The regression analysis should predict malaria risk using the haematological parameters as independent variables, and correcting for potential confounders such as age and possibly the study site, in order for the analysis to directly answer the research question.
- Direct comparison of infected with uninfected persons for all data assumes that the different transmission settings will have no impact on outcomes. This analysis should be done per site before combining all data. 
- Also, the table legend describes the study results. The legend should only describe how the information in the table was generated, not the results/data. Kindly remove all results from the legend. Figure 1 legend is a perfect example, with no data in legend, just a description of how the figure was created.
- Figure 1 needs the horizontal axis title. The vertical axis has parasites/ul

Is the work clearly and accurately presented and does it cite the current literature?

Partly

If applicable, is the statistical analysis and its interpretation appropriate?

Partly

Are all the source data underlying the results available to ensure full reproducibility?

Partly

Is the study design appropriate and is the work technically sound?

No

Are the conclusions drawn adequately supported by the results?

No

Are sufficient details of methods and analysis provided to allow replication by others?

Partly

Reviewer Expertise:

Immunology of infectious diseases, vaccine discovery and development

I confirm that I have read this submission and believe that I have an appropriate level of expertise to confirm that it is of an acceptable scientific standard, however I have significant reservations, as outlined above.

# Response

**Published:** 2020-07-09

**Authors:** Kingsley Badu

**Affiliations:** Kwame Nkrumah University of Science and Technology, Ghana

We have responded to all the concerns raised by the reviewer. The major changes made in the manuscript are;

1. We provide information on the ethical committee and approval number.

The Committee on Human Research Publication and Ethics of the Kwame Nkrumah University of Science and Technology and the Komfo Anokye Teaching Hospital (CHRPE/KNUST), reviewed and approved the study protocol (Ref: CHRPE AP/018/18).

2. We have re-organized Table 1, which now shows the statistical comparison amongst the three sites for each parameter.

3. We have included Figure 1 horizontal axis title.

Thank you

# Peer review: Reviewer response for version 1

**DOI:** 10.21956/aasopenres.14060.r27312

**Published:** 2020-01-27

**Authors:** Deus S. Ishengoma

**Affiliations:** 1 Tanga Research Centre, National Institute for Medical Research (NIMR), Tanga, Tanzania

**General comments:**

 The study aimed at assessing haematological changes among malaria patients residing in three different types of settlements. However, the study design and data collection methods could not be tailored to this overall aim. Thus, the manuscript needs major revisions before it can be accepted for publication.

**Specific comments:**

*Abstract:*

1. The design of this study cannot allow to investigate the changes in haematological parameters but rather to assess the status of the parameters at the time of the study. The first statement should be changed to take into account this fact.
2. I would recommend to change the way the results are presented in the abstract:
   
  1. The prevalence of malaria infection in the three settlements should be mentioned.
  2. The results of the analyses which compared malaria infected and non-infected patients should state which group had higher or lower haematological values rather that stating that there was a significant difference. This does not provide useful and adequate information to the readers.
  3. The results need to be reorganized to provide a more local flow of the findings. It may be useful to first compare all haematological parameters (overall) among infected vs non-infected patients; and then presenting the same values in infected (with or without symptoms) and non-infected asymptomatic patients from the three settlements.
3. The last statement in the abstract which states that, “Atypical results from routine haematological findings of anaemia and thrombocytopaenia, may be indicative of malaria, …” is not supported by the results presented in the abstract. Furthermore, it is generally known that malaria infection (particularly severe malaria) is associated with anaemia and thrombocytopaenia. This statement needs to be revised, otherwise it can be dropped.

*Introduction:*

1. The authors should use the data presented in the most recent WHO report of 2019, and should also note that the report normally presents data of the past year. For example, WHO report of 2019 contains malaria data of 2018 and not the same year (2019).
2. In the second paragraph, the authors mentioned that infection with malaria parasites trigger haematological changes. Apart from providing some citations, they could not give a brief account of those changes. It is important to provide a reader with the type of changes and the mechanisms associated with such changes.

*Methods:*

1. This was a hospital-based study which targeted patients with symptoms of malaria but later the investigators decided to include asymptomatic relatives. Was this the original plan or it was adopted just to avoid bias?
2. What were the inclusion and exclusion criteria?
3. How were patients sampled and recruited? Did the investigators target all patients attended at the study hospitals?
4. When was the study conducted?
5. Study sites: how and why were sites selected for this study? What criteria were used to select the hospitals?
6. What were the justifications/rationale for using the formula presented on page 3 and malaria prevalence in determining the sample size? I think that the expected differences in haematological parameters among the three settlements should have been taken into account in determining the sample size.
7. How were demographic and clinical data collected? Who collected the data? And what tools were used and how was quality control of the data performed?
8. Since some haematological data are significantly influenced by environment factors (e.g. altitude) and nutrition, were these data collected? If not, why?
9. I would recommend to re-analyse the data to determine the impact of malaria on haematological parameters by linear/logistic regression or similar analysis to determine the potential contribution of malaria infection on different parameters with adjustment for different co-variates such as age, sex, residence etc.

*Results:*

1. I would recommend presenting a baseline table showing key demographics (sex, age, ITNs use), prevalence and parasite density in the three study settlements.
2. The results show that 75.5% of the participants were female. Was this deliberately planned to collect more samples from female participants?  Due to the impact of sex on same parameters, this should have been avoided from the study design and sampling.
3. The age differences of participants were also different among participants from the three settlements. This should have also been avoided in the study design in order to get comparable groups.
4. I would recommend analysing the variations and impact of ITNs, malaria prevalence, age and settlement using logistic regression rather than χ ^2^-test.
5. The results comparing different parameters in symptomatic and asymptomatic participants should also be presented.
6. On page 4, the authors stated that they tested for the relationship between anaemia and thrombocytopaenia in malaria-infected patients. However, it is not mentioned in the method how this was done. It is important to have details in the data analysis section to show how the different tests were done.

*Discussion and conclusion:*

1. The authors mentioned that they investigated haematological changes in malaria patients in three settlements. However, the nature and design of this study cannot allow to assess the trends and changes in such parameters, which can better be assessed in a well-designed longitudinal study; with a design such as cohort or case-control study.
2. The differences in haematological parameters reported in both infected and non-infected participants in the three settlements should be thoroughly discussed. The possible explanations for such differences must be provided.
3. The study had many limitations in terms of design (targeted symptomatic patients but later switched to collecting asymptomatic care-givers as well), selection criteria (biased age and sex of patients), lack of data on altitude of residence, socio-economic and nutritional status, and other factors with significant impacts on haematological parameters. These and other limitations need to be well reported and taken into account when drawing the conclusion of the study.

*Other comments **.***

1. Under figure 1, it is mentioned that analysis was done using R software but this was not mentioned in the data analysis section.
2. The authors should revise the document to take care of typos.

Is the work clearly and accurately presented and does it cite the current literature?

Partly

If applicable, is the statistical analysis and its interpretation appropriate?

No

Are all the source data underlying the results available to ensure full reproducibility?

Partly

Is the study design appropriate and is the work technically sound?

No

Are the conclusions drawn adequately supported by the results?

Partly

Are sufficient details of methods and analysis provided to allow replication by others?

No

Reviewer Expertise:

Antimalarial drug resistance and Genomic epidemiology of malaria

I confirm that I have read this submission and believe that I have an appropriate level of expertise to confirm that it is of an acceptable scientific standard, however I have significant reservations, as outlined above.

# Response

**Published:** 2020-06-05

**Authors:** Kingsley Badu

**Affiliations:** Kwame Nkrumah University of Science and Technology, Ghana

**Response to reviewers’ comments**

We are very grateful for the comments provided by the reviewers of this manuscript. Please see below for our responses.

- ***Abstract:*** The design of this study cannot allow to investigate the changes in haematological parameters but rather to assess the status of the parameters at the time of the study. The first statement should be changed to take into account this fact.

**Response:** *We appreciate the reviewer’s comment and agree that investigating changes in haematological parameters was outside the scope of this study. We have changed the wording to better reflect the actual aims of the study*

- I would recommend to change the way the results are presented in the abstract:
  - The prevalence of malaria infection in the three settlements should be mentioned. 

**Response:** *We have now included this information in the abstract.*

- The results of the analyses which compared malaria infected and non-infected patients should state which group had higher or lower haematological values rather than stating that there was a significant difference. This does not provide useful and adequate information to the readers.

**Response:** *We have revised the text to include information on which group had higher or lower haematological values.*

- The results need to be reorganized to provide a more logical flow of the findings. It may be useful to first compare all haematological parameters (overall) among infected vs non-infected patients; and then presenting the same values in infected (with or without symptoms) and non-infected asymptomatic patients from the three settlements.

**Response:** *We have re-organised the results presented in the abstract to provide a more logical flow of the main findings of the study.*

 

- The last statement in the abstract which states that, “Atypical results from routine haematological findings of anaemia and thrombocytopaenia, may be indicative of malaria, …” is not supported by the results presented in the abstract. Furthermore, it is generally known that malaria infection (particularly severe malaria) is associated with anaemia and thrombocytopaenia. This statement needs to be revised, otherwise it can be dropped.

**Response:** *We have ‘dropped’ the statement in line with the reviewer’s suggestion.*

***Introduction:***

- The authors should use the data presented in the most recent WHO report of 2019, and should also note that the report normally presents data of the past year. For example, WHO report of 2019 contains malaria data of 2018 and not the same year (2019).

**Response:** *We have revised the manuscript to reflect the most recent malaria data available from the WHO.*

- In the second paragraph, the authors mentioned that infection with malaria parasites trigger haematological changes. Apart from providing some citations, they could not give a brief account of those changes. It is important to provide a reader with the type of changes and the mechanisms associated with such changes.

**Response:** *We have now provided a brief account of the most common haematological changes associated with malaria.*

*Methods:*

- This was a hospital-based study which targeted patients with symptoms of malaria but later the investigators decided to include asymptomatic relatives. Was this the original plan or it was adopted just to avoid bias? 

**Response:** *The study recruited all study participants at the beginning of the study.*

- What were the inclusion and exclusion criteria?

**Response:** *Since the study was carried out in the hospital laboratories, prospective participants were all who had been referred to the hospital laboratory for tests to be conducted, as well as any family members or friends who accompanied them. Babies younger than six months, patients who were critically ill and those dissenting to give their consent were excluded from the study. We have revised the text to make this information clearer.   *

- How were patients sampled and recruited? Did the investigators target all patients attended at the study hospitals?

**Response:** *We targeted all patients who had been referred to the hospital’s laboratory, together with any accompanying persons. And of-course only those who gave consent to participate*

- When was the study conducted?​​​​​​​

**Response:** *The study was conducted from January to December 2018.*

- Study sites: how and why were sites selected for this study? What criteria were used to select the hospitals?​​​​​​​

**Response:** *We selected the hospitals based on our knowledge of the malaria transmission cline. This ranges from,  AGH – moderately high; KGH – moderate; KSH – relatively low*

- What were the justifications/rationale for using the formula presented on page 3 and malaria prevalence in determining the sample size? I think that the expected differences in haematological parameters among the three settlements should have been taken into account in determining the sample size.

**Response:** *Unfortunately, there isn’t any prior information on differences in haematological parameters of residents of the three settlements. However, the malaria prevalence used was from a study that sought to predict malaria infection using the haematological parameters of patients from a hospital in a nearby town that can be classified as a peri-urban community. *

- How were demographic and clinical data collected? Who collected the data? And what tools were used and how was quality control of the data performed?

**Response:** *The data, that is to say, the questionnaire were administered by the authors of this article and not by research assistants. Authors are very experienced in conducting similar surveys from different parts of the country and in other research projects. At the end of everyday all researchers came together to check the completeness of each individual questionnaire and match them to the lab specimen taken. Prior to the survey we had pre-tested the questionnaire in a community setting to ensure people understood and that questions were effective. Feedback were used to improve the questionnaire before the actual survey was carried out. *

- Since some haematological data are significantly influenced by environment factors (e.g. altitude) and nutrition, were these data collected? If not, why?

**Response:** *We appreciate the reviewer’s comments on altitude and nutrition. However altitude will not be of any effect in our study sites, all of which are between 250m to 300m asl. Unlike the mountainous areas many parts of Kenya and Tanzania, Ghana is relatively flat. We did not collect information on other variable such as nutrition or intestinal helminths and others. We have been studying these communities for a while, we have other studies that focus on nutrition and other infections. However, this study focused on malaria. We do not expect the influence of ‘other environmental’ parameters to significantly differ in the population.*

 

- I would recommend to re-analyse the data to determine the impact of malaria on haematological parameters by linear/logistic regression or similar analysis to determine the potential contribution of malaria infection on different parameters with adjustment for different co-variates such as age, sex, residence etc.

**Response:** *We have re-analysed the data using logistic regression and believe the results are now easier to interpret (Tables 2 & 4).*

*Results:*

- I would recommend presenting a baseline table showing key demographics (sex, age, ITNs use), prevalence and parasite density in the three study settlements.

**Response:** *We appreciate the opportunity to include any additional information that makes our data easier to follow. We have now included a baseline table of demographic information (Table 1).*

- The results show that 75.5% of the participants were female. Was this deliberately planned to collect more samples from female participants? Due to the impact of sex on same parameters, this should have been avoided from the study design and sampling. 

**Response:** *We appreciate the concern raised by the reviewer. The main aim of the study was to assess the status of the haematological parameters. At the time of recruitment, these were the respondents who agreed to take part in the study after they were taken through the consenting process hence the high numbers of female participants were not deliberately planned. This notwithstanding, we entirely agree that gender category should ideally be as balanced it is practicable.*

- The age differences of participants were also different among participants from the three settlements. This should have also been avoided in the study design in order to get comparable groups.

**Response:** *This is a cross sectional study, where individual sent to the lab were approached for consent, we could not discriminate among willing participants. The age groups presented are study participants there were presented at the hospital and consented to participate. That said, future studies will take this into account.*

 

- I would recommend analysing the variations and impact of ITNs, malaria prevalence, age and settlement using logistic regression rather than χ -test.

**Response:** *We are very grateful to the reviewer for this suggestion. We have now re-analysed the data using logistic regression (Tables 2 & 4).*

- The results comparing different parameters in symptomatic and asymptomatic participants should also be presented.

**Response:** *The only available infection categories were the malaria-positive and malaria negative groups. We intended to further categorise the study participants into symptomatic and asymptomatic based on temperature readings. Unfortunately, our temperature readings could not reliably distinguish between the two categories. This is a limitation of this study.*

- On page 4, the authors stated that they tested for the relationship between anaemia and thrombocytopaenia in malaria-infected patients. However, it is not mentioned in the method how this was done. It is important to have details in the data analysis section to show how the different tests were done.

**Response:** *We have revised this section and completely removed the analysis on the relationship between anaemia and thrombocytopaenia in malaria-infected patients.*

*Discussion and conclusion:*

- The authors mentioned that they investigated haematological changes in malaria patients in three settlements. However, the nature and design of this study cannot allow to assess the trends and changes in such parameters, which can better be assessed in a well-designed longitudinal study; with a design such as cohort or case-control study.

**Response:** *We have re-worded the statements to reflect reporting on the status of the hematological parameters, not necessarily the ''changes'.*

- The differences in haematological parameters reported in both infected and non-infected participants in the three settlements should be thoroughly discussed. The possible explanations for such differences must be provided.

**Response:** *We discuss possible reasons for differences in haematological parameters between all infected and non-infected participants. However, it is now apparent that “settlement” is not significantly associated with malaria positivity. It is, correlated with “age” and this is likely the reason for the positive result using the χ ^2^–test.*

- The study had many limitations in terms of design (targeted symptomatic patients but later switched to collecting asymptomatic care-givers as well), selection criteria (biased age and sex of patients), lack of data on altitude of residence, socio-economic and nutritional status, and other factors with significant impacts on haematological parameters. These and other limitations need to be well reported and taken into account when drawing the conclusion of the study.

**Response:** *We thank the reviewer for bringing up this very important point. We have added a brief section on the limitations of this study towards the end of the manuscript. However, we maintain that 1. Altitude is not an issue in our study settings at it is in Tanzania and other East African altitudinal transects. The socio-economic and nutritional status are NOT severe confounding factors as in other studies we know they are similar in the populations.*

*Other comments* ***.***

- Under figure 1, it is mentioned that analysis was done using R software but this was not mentioned in the data analysis section.

**Response:** *This has been stated appropriately. *

- The authors should revise the document to take care of typos.

**Response:** *Typos have been taken care of.*

# Peer review: Reviewer response for version 1

**DOI:** 10.21956/aasopenres.14060.r27177

**Published:** 2019-09-18

**Authors:** Kwadwo Asamoah Kusi

**Affiliations:** 1 Department of Immunology, Noguchi Memorial Institute for Medical Research (NMIMR), University of Ghana, Legon, Ghana

The manuscript by Mutala *et al.* assesses malaria-related haematological changes in three different transmission zones and reports statistically significant differences in some of the measured indices between malaria infected and uninfected persons, and higher levels of some indices in infected persons in peri-urban areas compared to levels in persons in the rural and urban areas.

 The paper will require some significant revision to better communicate the study findings.

**Major comments:**

1. Authors describe the three study locations as rural, peri-urban and urban. It is unclear from the write up why it is necessary to compare these three demographic settings for haematological differences. If this is being done because of possible differences in transmission intensity however, then it may be more appropriate for the authors to present some current malaria transmission data from these sites and on that basis rather, proceed to refer to these as different transmission intensity settings.
2. Authors should clearly state whether this is a cohort or case control study. Authors should also describe any exclusion criteria that were applied during sampling as the conclusions of the study and potential application of the data may only be valid if certain exclusion criteria were applied. There are some common conditions/infections that could also impact these haematological indices, and without accounting for these, it may not be appropriate to conclude that the observed differences in the indices are due to malaria infection.
3. The manner of results presentation makes comprehension difficult. Authors refer to all 598 participants whose samples were analyzed as patients at some times (second paragraph of the results section), and even though on the basis of the study design, participants can be categorized into those who reported clinically sick, those with asymptomatic infections and those who may be uninfected (by microscopy), no such breakdown in terms of age within these three groups is given in the first subsection of the results. For example, how many of the persons accompanying sick people to the hospital were parasite positive?
4. Most of the subsequent comparisons refer to an infected group and a control group, and it is unclear whether the infected group is in reference to the clinically ill group alone or a combined group of clinically ill and asymptomatic persons. If the latter, what was the reason for combining these two distinct groups as they are likely to have very different haematological profiles?
5. The age range of study participants is not provided. The youngest age groups is given as 0 – 5 years (Table 2).
6. Table 1 seems to be a comparison between infected and uninfected participants from all 3 study sites, but if the goal of the study was to assess differences across different settlements, then why is it relevant to combine data from the three study sites and compare between infected and uninfected persons? A combined presentation of information in Table 1 and Table 3 will be more consistent with the aim of this study, to compare infected and uninfected (maybe sick and not sick rather) for each community.
7. Overall, the data presentation needs to be overhauled. That aside, the presented data does not justify the conclusion that changes in haematological indices can be used as indicative factors for malaria, especially when they depended on the community in which they were measured. I would agree with authors if the indices were consistently different between sick and not-sick persons in all three study sites. Moreover, the unavailability of exclusion criteria in this write up makes it difficult to agree that these observations are due to malaria infections.

**Minor comments:**

1. Abstract, penultimate line before the conclusions section – “…levels amongst patients from urban, …”, not “…between patients…”.
2. “Thrombopaenia”, not “thrombopenia”, to be consistent with all other British spelling of words (parasitaemia, haematological).
3. Authors should provide the protocol approval number.
4. The statistical analysis section should be updated to reflect the fact that the R software was also used for some graphics (Figure 1).
5. Second sentence of the results section should read: “In total, 598 participant samples…”, not “…participants samples…”.
6. “Kruskal-Wallis”, not “Kruskall-Wallis” (statistical analysis section, Legend to Table 3).
7. Discussion section, lines 5/6 – which group do the authors refer to as non-malaria patients?

Is the work clearly and accurately presented and does it cite the current literature?

Partly

If applicable, is the statistical analysis and its interpretation appropriate?

Partly

Are all the source data underlying the results available to ensure full reproducibility?

Partly

Is the study design appropriate and is the work technically sound?

No

Are the conclusions drawn adequately supported by the results?

No

Are sufficient details of methods and analysis provided to allow replication by others?

Partly

Reviewer Expertise:

Immunology of infectious diseases, vaccine discovery and development

I confirm that I have read this submission and believe that I have an appropriate level of expertise to state that I do not consider it to be of an acceptable scientific standard, for reasons outlined above.

# Response

**Published:** 2020-06-05

**Authors:** Kingsley Badu

**Affiliations:** Kwame Nkrumah University of Science and Technology, Ghana

**We are very grateful to the reviewer, his comments has enabled us clarify and improve the quality of the manuscript. Here we provide point by point response to the comments raised by the reviewer.**

**Major Comments**

- Authors describe the three study locations as rural, peri-urban and urban. It is unclear from the write up why it is necessary to compare these three demographic settings for haematological differences. If this is being done because of possible differences in transmission intensity however, then it may be more appropriate for the authors to present some current malaria transmission data from these sites and on that basis rather, proceed to refer to these as different transmission intensity settings.

**Response:** *We thank the reviewer for this comment. It makes more biological sense to compare on the basis of transmission. We have provided background information on the malaria transmission intensity and associated references in the manuscript. Whereas people in the Agona area ( we describe as rural and high transmission ) receive over 11,000 mosquitoe bites with over 800 infectious bites, the Kumasi area receives about 400 bites and about 200 infectious bite per year. The Kuntanase area is moderate in between the two [Abonuusum et al 2011, and Basing et al 2014] The citations are below* 

- Authors should clearly state whether this is a cohort or case control study. Authors should also describe any exclusion criteria that were applied during sampling as the conclusions of the study and potential application of the data may only be valid if certain exclusion criteria were applied. There are some common conditions/infections that could also impact these haematological indices, and without accounting for these, it may not be appropriate to conclude that the observed differences in the indices are due to malaria infection.

**Response:** *We thank the reviewer for bringing up this very important point. This was an observational study with cross-sectional sampling design in which participants were recruited from three hospitals located in different transmission intensity settings. Since participants were recruited from hospitals, we referred to them as “patients”; however, it is now apparent that this term is misleading so we have removed most references to “patients” from the manuscript. We are also aware that many viral and bacterial infections present with malaria-like symptoms, and may also affect haematological indices similarly. This is one of the limitations of our study.* 

- The manner of results presentation makes comprehension difficult. Authors refer to all 598 participants whose samples were analysed as patients at some times (second paragraph of the results section), and even though on the basis of the study design, participants can be categorized into those who reported clinically sick, those with asymptomatic infections and those who may be uninfected (by microscopy), no such breakdown in terms of age within these three groups is given in the first subsection of the results. For example, how many of the persons accompanying sick people to the hospital were parasite positive?

**Response:** *We have re analysed the data based on reviewer’s comments also echoed by Reviewer 1.*

*We believe the data is now much more clearer to understand. We refer to two categories as malaria infected and not infected due to challenges in distinguishing between symptomatic and asympotmatics earlier described *

- Most of the subsequent comparisons refer to an infected group and a control group, and it is unclear whether the infected group is in reference to the clinically ill group alone or a combined group of clinically ill and asymptomatic persons. If the latter, what was the reason for combining these two distinct groups as they are likely to have very different haematological profiles?

**Response:** *The infected group refers to those who tested positive for malaria, regardless of symptoms. We pooled symptomatic and asymptomatic participants together as there was no other way to distinguish between the two groups due to problems with our temperature readings.*

- The age range of study participants is not provided. The youngest age groups is given as 0 – 5 years (Table 2). 

**Response:** *The age range of study participants has been presented in Table 1. Participants were grouped into three categories: 0—5 years, 6—14 years and 15+ years.*

- Table 1 seems to be a comparison between infected and uninfected participants from all 3 study sites, but if the goal of the study was to assess differences across different settlements, then why is it relevant to combine data from the three study sites and compare between infected and uninfected persons? A combined presentation of information in Table 1 and Table 3 will be more consistent with the aim of this study, to compare infected and uninfected (maybe sick and not sick rather) for each community.

**Response:** *We are grateful for this comments from the reviewer to improve our manuscript.*

*After re-analysing the data using logistic regression, it is clear that “settlement” is not significantly associated with malaria positivity. It is, however, correlated with “age” and this is likely the reason for the positive result using the χ ^2^–test. As settlement is no longer a predictor of malaria positivity, we decided to completely remove Table 3.*

- Overall, the data presentation needs to be overhauled. That aside, the presented data does not justify the conclusion that changes in haematological indices can be used as indicative factors for malaria, especially when they depended on the community in which they were measured. I would agree with authors if the indices were consistently different between sick and not-sick persons in all three study sites. Moreover, the unavailability of exclusion criteria in this write up makes it difficult to agree that these observations are due to malaria infections.

**Response:** *Our data presentation has been ‘overhauled' as recommended by reviewers, data has been re analyzed. Thus the conclusion has been subsequently re-worded and we have included the exclusion criteria in the methods section as requested.*

**Minor comments:**

- Abstract, penultimate line before the conclusions section – “…levels amongst patients from urban, …”, not “…between patients…”. 

**Response:** *We thank the reviewer for this comment. Sentence corrected as suggested. *

- “Thrombopaenia”, not “thrombopenia”, to be consistent with all other British spelling of words (parasitaemia, haematological). 

**Response:** *We thank the reviewer for this observation. The manuscript has been revised to effect these changes.  *

- Authors should provide the protocol approval number. 

**Response:** *The approval number has been included in the manuscript.*

- The statistical analysis section should be updated to reflect the fact that the R software was also used for some graphics (Figure 1). 

**Response:** *This has been included in the revised manuscript. *

- Second sentence of the results section should read: “In total, 598 participant samples…”, not “…participants samples…”. 

**Response:** *The proposed correction has been effected in the manuscript. *

- “Kruskal-Wallis”, not “Kruskall-Wallis” (statistical analysis section, Legend to Table 3). 

**Response:** *This correction has been effected in the manuscript. Thank you.*

- Discussion section, lines 5/6 – which group do the authors refer to as non-malaria patients? 

**Response:** *The sentence has been modified in the manuscript.* 

Abonuusum A, Owusu-Daako K, Tannich E, May J, Garms R, Kruppa T. Malaria transmission in two rural communities in the forest zone of Ghana. Parasitol Res. 2011;108(6):1465‐1471. doi:10.1007/s00436-010-2195-1

A.W. Basing∗, S. TayMalaria transmission dynamics of the Anopheles mosquito in Kumasi, Ghana 16th ICID Abstracts / International Journal of Infectious Diseases 21S (2014) 1–460
